# Wild honey poisoning: A case report from western Nepal

**DOI:** 10.1002/ccr3.5527

**Published:** 2022-03-01

**Authors:** Anuj Krishna Paudel, Amrit Pokhrel, Suman Gaire, Ananta Hari Paudel, Sujan Poudel, Suman Paudel, Wilson Cueva, George J. Michel

**Affiliations:** ^1^ Intensive Care Unit (ICU) Metrocity Hospital and Research Center Pokhara Nepal; ^2^ Emergency Department Metrocity Hospital and Research Center Pokhara Nepal; ^3^ Emergency Department Palpa Hospital Palpa Nepal; ^4^ Larkin Community Hospital South Miami Florida USA

**Keywords:** bradycardia, grayanotoxin, hypotension, wild honey

## Abstract

Wild honey has been used for several purposes in South‐Asia and Eastern Europe since long ago. One of the commonest is medicinal purposes, especially for gastrointestinal disorders (peptic ulcer disease, dyspepsia, and gastritis), hypertension, and an aphrodisiac (sexual stimulant). However, honey produced from the nectar of few rhododendron species contains a toxin known as grayanotoxin, which acts on the sodium channel and prevents its inactivation leading to vagal activation, causing hypotension and bradycardia. Here, we report a case of 55 years woman who reported to our Emergency Department with complaints of dizziness and vomiting as well as bradycardia and hypotension. Most of the cases of wild honey poisoning resolve within the first 24 h, but in our case, the symptoms persisted for 72 h. So, we must observe patients for 72 h as some cases may last longer. Timely management of the patient can prevent fatal complications.

## Graphical abstract

Most cases of wild honey poisoning resolve within the first 24 h, but in some cases, the symptoms may persist for up to 72 h. So, we must observe patients for at least 72 h.

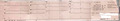

## 1 INTRODUCTION

Wild (Mad) honey poisoning is due to ingestion of honey containing a toxin known as grayanotoxin, which is also known as rhodotoxin because it is derived from the nectar of a few rhododendron species primarily found in the mountainous region of Bhutan, India, Nepal, and Turkey.^1^ Mad honey is used as an alternative medicine for hypertension, diabetes, flu, gastrointestinal disorders (peptic ulcer, gastritis, dyspepsia, indigestion, and bowel disorder), and arthritis, stimulating sex (dysfunction, impotence, enhancement, and performance), viral infections, and cold.^2^ Mad honey can cause dramatic side effects such as nausea, vomiting, dizziness, sweating, impaired consciousness, and various cardiac side effects like bradycardia and hypotension.^3^ Ingestion honey containing grayanotoxin can result in hypotension, bradycardia, and cardiac rhythm disorders (first‐, second‐, and third‐degree AV block, asystole, and sinus bradycardia).^4^, ^5^ Here, we present a case of mad honey poisoning, which developed bradycardia, hypotension, and dizziness.

## 2 CASE REPORT

**FIGURE 1:**
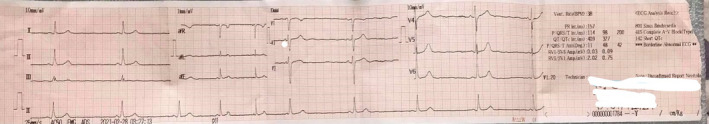
ECG of the patient on admission, Heat rate 50 bpm

A 55‐year‐old woman presented to the emergency department with complaints of sudden onset of dizziness and vomiting for 2 h. Further history revealed that the symptoms appeared after 45–60 min after ingestion of 10–15 ml of wild honey. There was no history of trauma, alcohol, or drugs. The patient was a known case of hypertension under treatment with Losartan 25 mg per orally once daily for 3 months. On examination, the patient was oriented to time, place, and person. Her blood pressure was 70/40 mm hg, pulse rate was 48 beats per minute and irregular, temperature 98.7°F (37.05°C), respiratory rate 16/min, and oxygen saturation was 97% at room air. Immediate resuscitation was done with intravenous(IV) fluids and IV hydrocortisone 100 mg. 12 lead ECG showed sinus bradycardia with 50 per minute of heart rate, as shown below in the ECG (Figure 1). Blood investigations were within the normal limits as shown in Table 1.

**TABLE 1:** Blood Investigations

Blood tests	Values
Hemoglobin (12–16 g/dl)	11.4
Random blood sugar (60–200 mg/dl)	96
Serum Na^+^ (135–145 mmol/L)	138
Serum K^+^ (3.5–5.5 mmol/L)	4
Thyroid‐stimulating hormone (0.4–4.2 mIU/L)	2.1
Serum calcium (8.5–11 mg/dl)	8.4
Serum creatinine (0.3–1.1 mg/dl)	1

**FIGURE 2:**
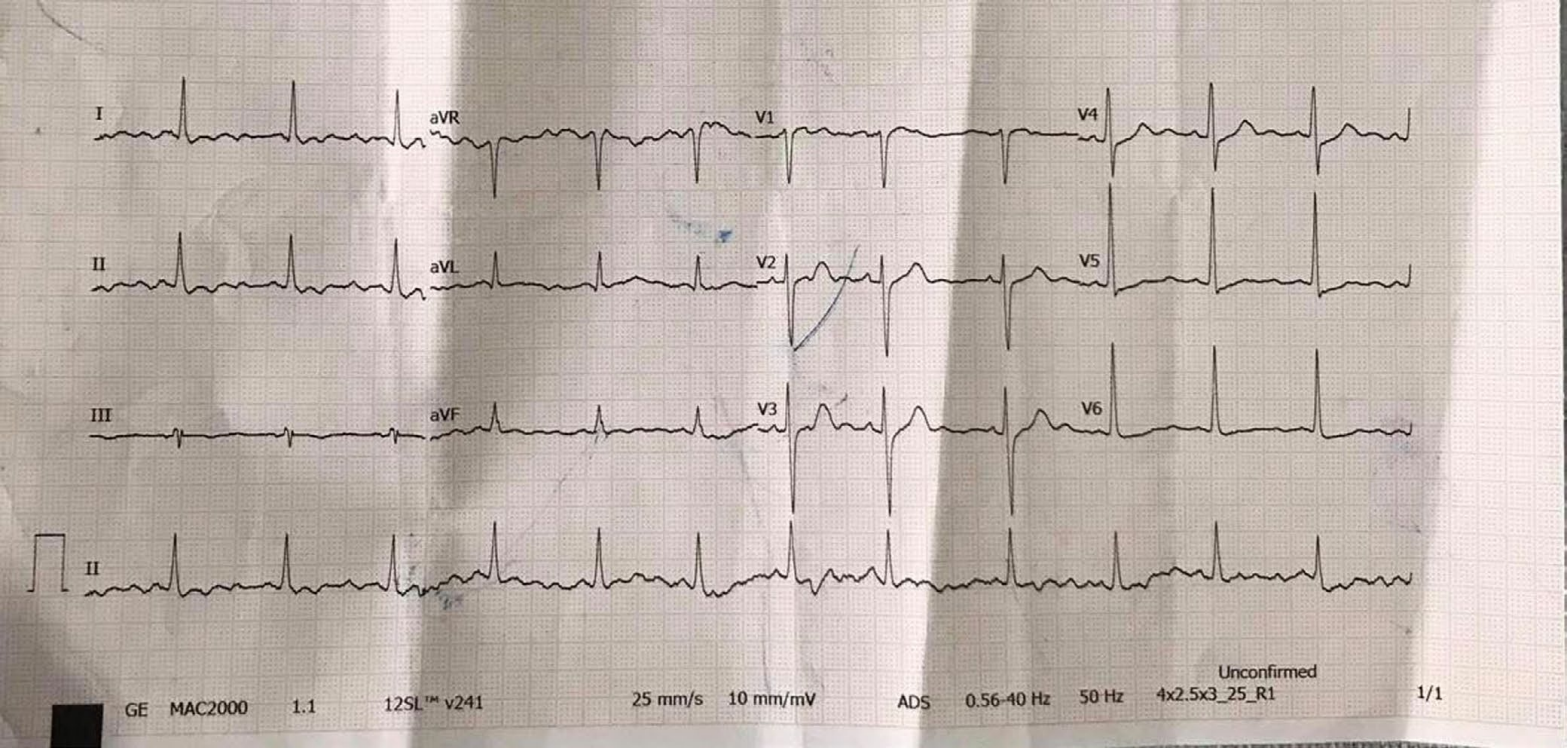
ECG of the patient at Discharge, Heart Rate 86 bpm

Subsequently, the patient was admitted to the ICU, where the patient was kept under constant monitoring with normal saline, IV hydrocortisone 100 mg three times a day, and IV atropine if required (heart rate < 40 bpm). After administering 2000 ml (1000 ml in Emergency and 1000 ml in ICU) of Normal saline, her blood pressure reached up to 90/60 mm Hg with a pulse rate of 58 beats/minute. After 12 h of admission to ICU, her blood pressure was gradually stabilized to 120/80 mm Hg with a heart rate of 72 beats per minute. The patient did not require vasopressor support. The patient was kept under cardiac monitoring in the intensive care unit and was discharged after 24 h of admission into ICU on patient request. After 12 h of discharge from the hospital patient again presented to the hospital complaining of dizziness and weakness. On examination, her heart rate had decreased to 52 beats per minute, but her blood pressure was within the normal range. She was again admitted into ICU under constant cardiac supervision with maintenance fluid and hydrocortisone 100 mg three times a day and atropine if required (heart rate < 40 bpm). She was then discharged 36 h after second admission into the ICU with blood pressure 130/70 mm of Hg and a heart rate of 86 beats per min, as shown in Figure 2.

## 3 DISCUSSION

Grayanotoxin is found in the pollens of the flower Rhododendron. There are 18 types of grayanotoxin found, out of which grayanotoxin 1 and 2 have been found in the honey, leaves, and flowers of Rhododendron, which are considered the most potent form of toxins.^1^ In a study of clinical cases, the minimum blood level of grayanotoxin to cause hypotension was reported between 2.52 and 4.55 ng/ml.^6^ A direct comparison of the amount of grayanotoxin in honey found across the world has not been done. However, in the case of mad honey poisoning in France from honey taken from Nepal, the level of grayanotoxin 1 in Nepalese honey was measured. The author reported the levels to be higher than normal levels of grayanotoxin 1 in Turkish honey.^7^

The toxin binds to the voltage‐gated sodium channel preventing sodium channel inactivation, causing depolarization and ultimately leading to vagal activation, inducing bradycardia and hypotension.^5^ Cholinergic symptoms are present in the majority of the patients, but life‐threatening cardiac complications occur in a few. In a systematic review of 1199 cases of mad honey poisoning, the most common presentation was with hypotension (19.75%). The most common ECG finding was sinus bradycardia seen in 79.58% of patients.^8^ Gastrointestinal symptoms like nausea, vomiting, neurological symptoms like blurring of vision, ataxic gait, peripheral numbness, and psychiatric symptoms have also been reported from mad honey poisoning in Nepal.^9^

Grayanotoxin levels can be measured in blood and urine. However, the facility to measure the toxin levels is not available in the many hospitals of Nepal including ours. Hence, we could not measure the levels of grayanotoxin in our patients. The history of honey ingestion becomes of utmost importance, especially in cases presenting with hypotension or bradycardia without other specific causes. Since the condition resolves in itself in the majority of cases with symptomatic treatment, it is likely many patients may not be identified if there is no high index of suspicion.

Most of the cardiac complications, if recognized on time, can be successfully managed with judicious use of IV fluids and atropine. Very rarely, transcutaneous cardiac pacing may be required.^2^

The onset of symptoms occurs few hours after ingestion of honey, and in most cases, the symptoms last for only 24 h.^10^ It has remained a general understanding that mad honey poisoning resolves by 24 h. However, in our case, the symptoms were persistent for up to 72 h. Because of this, the patient had to be readmitted and monitored in the intensive care unit. Therefore, we must be cautious and observe the patients with mad honey poisoning for 72 h. As the occurrence of mad honey poisoning is rare and occurs only in fixed geographical locations, further studies needed to be done to deepen our understanding of the disease.

## 4 CONCLUSION

Mad honey poisoning is rare and occurs in specific geographical locations. It may be difficult to diagnose the case if the index of suspicion is not high because of the lack of proper laboratory facilities to measure the level of grayanotoxin. History of ingestion of wild honey should be asked in patients presenting with hypotension and bradycardia if other causes are not found or if they resolve on their own. Although almost all cases of mad honey poisoning resolve within 24 h, in our patient, it was symptomatic for 72 h. So, the patients with mad honey poisoning should be monitored for 72 h.

## CONFLICT OF INTEREST

None.

## AUTHOR CONTRIBUTIONS

AKP involved in conceptualization of the case report, prepared the manuscript, edited the manuscript, and submission. AP collected the patient's information. SG prepared and reviewed literature. AHP collected the patient's information and prepared the manuscript. SP prepared the manuscript and reviewed the literature review. GM and WC prepared and edited the manuscript.

## ETHICAL APPROVAL

Written informed consent was obtained from the patient for publication of the case report.

## CONSENT

Written informed consent was obtained from the patient for publication of the case report.

## Data Availability

Data available on request from the authors.
